# Molecular subgrouping of medulloblastoma based on few-shot learning of multitasking using conventional MR images: a retrospective multicenter study

**DOI:** 10.1093/noajnl/vdaa079

**Published:** 2020-06-22

**Authors:** Xi Chen, Zhen Fan, Kay Ka-Wai Li, Guoqing Wu, Zhong Yang, Xin Gao, Yingchao Liu, Haibo Wu, Hong Chen, Qisheng Tang, Liang Chen, Yuanyuan Wang, Ying Mao, Ho-Keung Ng, Zhifeng Shi, Jinhua Yu, Liangfu Zhou

**Affiliations:** 1 Department of Electronic Engineering, Fudan University, Shanghai, China; 2 Department of Neurosurgery, Huashan Hospital, Fudan University, Shanghai, China; 3 Department of Anatomical and Cellular Pathology, The Chinese University of Hong Kong, Prince of Wales Hospital, Hong Kong, China SAR; 4 Department of Radiology, Huashan Hospital, Fudan University, Shanghai, China; 5 Department of Neurosurgery, Huadong Hospital, Fudan University, Shanghai, China; 6 Department of Neurosurgery, Shandong Provincial Hospital, Jinan, China; 7 Department of Pathology, the First Affiliated Hospital of USTC, Division of Life Sciences and Medicine, University of Science and Technology of China, Hefei, China; 8 Department of Pathology, Huashan Hospital, Fudan University, Shanghai, China

**Keywords:** few-shot learning, medulloblastoma, molecular subgrouping, MRI, prognosis categorization

## Abstract

**Background:**

The determination of molecular subgroups—wingless (WNT), sonic hedgehog (SHH), Group 3, and Group 4—of medulloblastomas is very important for prognostication and risk-adaptive treatment strategies. Due to the rare disease characteristics of medulloblastoma, we designed a unique multitask framework for the few-shot scenario to achieve noninvasive molecular subgrouping with high accuracy.

**Methods:**

We introduced a multitask technique based on mask regional convolutional neural network (Mask-RCNN). By effectively utilizing the comprehensive information including genotyping, tumor mask, and prognosis, multitask technique, on the one hand, realized multi-purpose modeling and simultaneously, on the other hand, promoted the accuracy of the molecular subgrouping. One hundred and thirteen medulloblastoma cases were collected from 4 hospitals during the 8-year period in the retrospective study, which were divided into 3-fold cross-validation cohorts (*N* = 74) from 2 hospitals and independent testing cohort (*N* = 39) from the other 2 hospitals. Comparative experiments of different auxiliary tasks were designed to illustrate the effect of multitasking in molecular subgrouping.

**Results:**

Compared to the single-task framework, the multitask framework that combined 3 tasks increased the average accuracy of molecular subgrouping from 0.84 to 0.93 in cross-validation and from 0.79 to 0.85 in independent testing. The average area under the receiver operating characteristic curves (AUCs) of molecular subgrouping were 0.97 in cross-validation and 0.92 in independent testing. The average AUCs of prognostication also reached to 0.88 in cross-validation and 0.79 in independent testing. The tumor segmentation results achieved the Dice coefficient of 0.90 in both cohorts.

**Conclusions:**

The multitask Mask-RCNN is an effective method for the molecular subgrouping and prognostication of medulloblastomas with high accuracy in few-shot learning.

**Key Points**

- Proposing a unique multitask framework for the few-shot learning scenario.
- Molecular subgrouping of medulloblastoma into 4 subtypes with an average AUC of 0.95.
- Simultaneous tumor segmentation and prognosis prediction.

**Importance of the Study**

In the latest version of the WHO 2016 classification of medulloblastoma, the molecular subgroups (WNT, SHH, Group 3, and Group 4) were widely recognized as the markers for patient treatment and prognosis strategies. Due to the technological complexity and cost, molecular subgrouping of medulloblastoma based on the genomic test might be unavailable in every neurosurgical center as routine clinical practice. Recently, radiomics method provided a potential tool for noninvasive molecular subgrouping of medulloblastoma. Regarding the relatively low incidence of medulloblastoma, our study designed a multitask network for the few-shot learning scenario with a small sample size by using preoperative MR images. Through the multitask technique, the proposed scheme achieved high diagnostic accuracy in both cross-validation and independent testing, with an average AUC of 0.97 and 0.92, respectively.

Medulloblastoma (MB) is the most common malignant pediatric brain tumor with about 40% being located in posterior cranial fossa.^1–3^ The 5-year survival rate reaches 65–70% in terms of tumor resection plus chemo-radiotherapy.^4^ Although the survival rates have increased dramatically during the past decade, the clinical outcome still varies a lot due to underlying biological distinctions.^5,6^ Based on genomic profiles, 4 distinct subgroups (wingless [WNT], sonic hedgehog [SHH], Group 3, and Group 4) were widely recognized and adapted into the latest version of WHO 2016 medulloblastoma classification.^7–10^ Tumors with different molecular subgroups have different clinical outcomes and chemo-radiosensitivity.^11–13^ Meanwhile, for surgery, the extent of surgical resection had no significant patient survival outcome, hence accounting for molecular subgroups.^4^ Thus, more and more studies have highlighted the importance of molecular subgrouping in the precise diagnosis and treatment of MB.^14–21^

Several laboratory methods have been developed to perform molecular subgrouping of MB by using tumor samples from surgical resection. Owing to technical complexity and costs, these methods were not applicable for routine clinical practice in a large number of medical facilities. However, the magnetic resonance (MR) scanning costs much less than the genomic test and could obtain medical images with high resolution. Moreover, this medical imaging method does not expose patients to ionizing radiation that causes tissue damage to patients. Hence, conventional MR images are first-hand clinical information for clinicians with cost-effective, high-resolution, and noninvasive traits. By analyzing tumor characteristic of MR images, several papers have been published to correlate the subgroups of MB with location, enhancing pattern, cystic change, and other image features reviewed by radiologists.^6,14–21^ Recently, radiomics has emerged as a new medical tool to bridge high-throughput MR features with tumor genomic and transcriptome profiling. With the help of deep learning techniques, radiomics methods have provided the possibility to perform accurate molecular diagnosis of various brain tumors such as glioma, metastases, and craniopharyngioma preoperatively and noninvasively.^22–24^

However, the data-driven predictions by deep learning techniques highly depend on a large number of training samples. The number of training samples limited the performance of radiomics algorithms in biomedical image analysis. The age-adjusted incidence rates per 100 000 person-years of MB are about 0.16 in all ages and about 0.44 in children (0–14 years).^2^ Very few papers were published about radiomics study in MB because of the limited number of cases. In our study, an improved multitask learning method for the predictive analysis of molecular subgroups of MB is designed based on the mask regional convolutional neural network (Mask-RCNN) to compensate the lack of training data.^25^ The multitask technique^26–28^ was introduced into radiomics modeling to reduce the risk of overfitting and to improve the generalization ability of facing the few-shot challenge. This method has been widely used in the field of brain tumor analysis and achieved impressive results. Liu et al.^29^ introduced the multitask technique jointly identifying the stage of Alzheimer’s disease and predicting clinical scores incorporating MR images and demographic information. Bui et al.^30^ proposed a multitask learning method to simultaneously learn both tissue segmentation and geodesic distance regression using brain MR images. Estienne et al.^31^ proposed a multitask algorithm that addresses the problems of image registration and brain tumor segmentation jointly. Collier et al.^32^ used a multitask learning strategy to integrate information about gene mutations and protein–protein interactions in a versatile manner and to predict tumor-driver genes in a pan-cancer setting and also for specific tumor types. Considering that location was proved to be an important MRI feature for molecular subgrouping of MB and the 4 molecular subgroups had different prognosis,^14,21,33^ the information of tumor mask and patient prognosis were integrated into the multitask framework to promote the accuracy of molecular subgroups prediction. Moreover, to effectively utilize the comprehensive information of our dataset, we expanded the input views of the Mask-RCNN. Both T1 contrast-enhanced (T1C) and T2 sequences were incorporated as input to obtain multi-view feature representations of patients. To the best of our knowledge, this is the first attempt at introducing the multitasking technique of few-shot learning to assist the preoperative prediction of molecular subgrouping in MB.

## Materials and Methods

### Patients Selection

This multicenter retrospective study was approved by the institutional ethics board of 4 participating hospitals (Huashan Hospital, Huadong Hospital, Shandong Provincial Hospital, and Anhui Provincial Hospital). We retrospectively reviewed the medical systems of 4 hospitals during the 8-year period (from January 2010 to December 2017) and screened out 120 patients diagnosed with MB histologically with high-quality preoperative MRI data and tumor tissue for molecular subtyping. The collected high-quality MR images have no artifacts and the diseased tissue could be clearly observed on them. Seven cases diagnosed as “medulloblastoma highly suspected” by neuropathologists were excluded because of very limited tumor samples. Finally, 113 patients were included from 4 hospitals in this study (Huashan Hospital = 52, Huadong Hospital = 22, Shandong Provincial Hospital = 16, and Anhui Provincial Hospital = 23). The detailed clinical and genomic characteristics of all cases from different institutions were given in Table 1. Seventy-three patients had a complete follow-up. Patients younger than 18 years were defined as child medulloblastomas, and those aged older than 18 years are defined as the adult counterparts. According to patients’ prognosis, they were also divided into 2 categories: good prognosis (overall survival [OS] time longer than 27 months) and poor prognosis (OS time shorter than 27 months). The OS time of 27 months was the median OS time of all patients in our study. Patient-informed consent was waived due to the retrospective study.

**Table 1.** Clinical and Genomic Characteristics of Different Institutions

Characteristic	Cross-validation cohort (*n* = 74)			Independent testing cohort (*n* = 39)			*P*
	Huashan (*n* = 52)	Huadong (*n* = 22)	Total (*n* = 74)	Shandong (*n* = 16)	Anhui (*n* = 23)	Total (*n* = 39)	
Molecular subgroup							.902
WNT	15	2	17	2	5	7	
SHH	11	7	18	5	4	9	
Group 3	13	7	20	3	8	11	
Group 4	13	6	19	6	6	12	
Age, years							.488
≤5		1	2	0	5	5	
6–18	29	11	40	11	12	23	
≥18		10	32	5	6	11	
Gender							.104
Male	38	15	53	10	12	22	
Female	14	7	21	6	11	17	
OS time							.382
≥27 months	33	5	38	7	13	20	
<27 months	19	17	36	9	10	19	
Histology							.101
Classic	28	16	44	9	5	14	
Desmoplastic	13	2	15	3	7	10	
Nodularity	2	2	4	0	6	6	
LCA	5	2	7	0	4	4	
N/A^a^	4	0	4	4	1	5	

OS, overall survival; LCA, large cell and/or anaplastic.

^a^Pathologic categories of 9 patients are difficult to judge due to the suboptimal quality of tumor tissues and denoted as N/A.

### Molecular Analysis

Molecular subgroup affiliation was done by the NanoString-based platform according to the previous publication.^34^ In brief, RNA was extracted from formalin-fixed paraffin-embedded (FFPE) tissues with an RNeasy FFPE Kit (Qiagen). RNA was then quantified by a NanoDrop 2000 spectrophotometer. A total of 100 ng RNA was then hybridized using the NanoString nCounter CodeSet at 67ºC for 20 h. The CodeSet contained gene-specific probes that assayed the abundance of 22 molecular-subgroup-specific genes and 3 housekeeping genes. Then, the excessive probes were washed, purified, and immobilized on a streptavidin-coated cartridge using the nCounter Prep Station (NanoString Technologies) according to the manufacturer’s protocol. Signals of fluorescent barcodes representing the target RNA molecules were then counted and recorded by the nCounter Digital Analyzer (NanoString Technologies). An automated script together with a training set was employed to transform raw data into subgroup prediction. The script normalized the raw data with R package NanoStringNorm, trained the pamr classifier with the training set, and made a subgroup prediction.

### MR Imaging

Brain MR imaging of enrolled patients was performed on MR systems, including Siemens, GE, and Philips, at 3 T before surgery to collect T1 contrast-enhanced (T1C) and T2 sequences. MR images were acquired using different protocols during the 8-year period. The protocol parameters were given in Supplementary Table S1. Two experienced radiologists blinded to patients’ clinical, pathologic, and molecular characteristics reviewed the imaging data and drew the region of interest around the tumor margin in MR images independently, and the final tumor segmentation results were confirmed by both radiologists.

### Mask Regional Convolutional Neural Network

**Figure 1.**
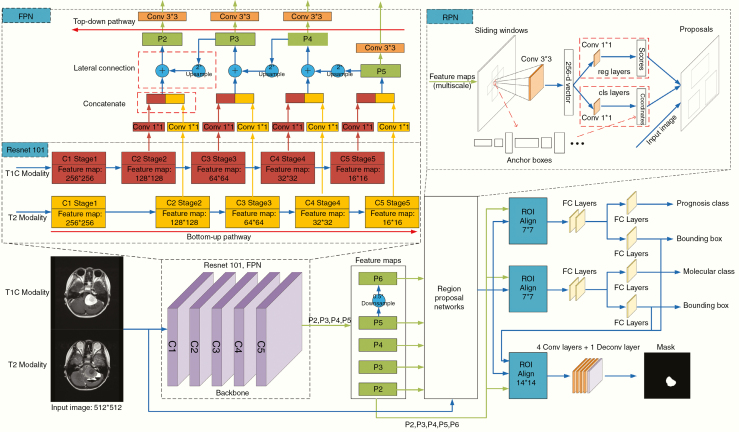
Illustration of our improved Mask-RCNN model.

Taking into consideration the low incidence of MB, we introduced a multitask technique to address the few-shot challenge. Due to the mechanism of exploiting the generic information shared across tasks and specific information among tasks, multitask learning could reduce the risk of overfitting and improve generalization ability and hence was efficient in few-shot scenarios.^26–28^ In our study, we adopted the Mask-RCNN as the original multitask framework and expanded the input views and the output tasks in the improved model. To take full advantage of our collected imaging data, we expanded our multitask model inputs with both T1C and T2 MRI sequences to obtain multiview feature representations. Meanwhile, 2 other related auxiliary tasks of prognosis categorization and tumor segmentation were tackled to constitute multitask learning in order to further improve the molecular diagnosis. The flowchart of our model is shown in Figure 1.

The improved Mask-RCNN model consisted of 3 stages: feature extraction, region proposal, and prediction. In the first stage, a feature pyramid network (FPN)^35^ with the refined basic feature extraction layers of Residual Neural Network (ResNet101)^36^ was first utilized as the backbone of the Mask-RCNN. The ResNet101 was comprised of 5 stages: C1, C2, C3, C4, and C5. By using pyramid representations to construct feature pyramids, multiscale features were extracted to combine the FPN with the ResNet101 in the study. FPN constructed 3 parts: a bottom-up pathway, a top-down pathway, and lateral connections. The feed-forward propagation of the ResNet101 was defined as the bottom-up pathway. The outputs of different stages were employed to form the feature maps of different pyramid levels. In the top-down pathway, the spatially coarser but semantically stronger feature maps from the higher pyramid levels were up-sampled by a factor of 2 to acquire higher resolution features. Then the up-sampled features from the top-down pathway were merged with the feature maps of the same spatial size from the bottom-up pathway by element-wise addition. Finally, each merged map was appended a 3*3 convolutional layer to acquire the final feature maps {P2, P3, P4, P5}. The feature map of P6 was obtained by subsampling P5 with a stride of 2. The pyramid feature maps were composed of P2, P3, P4, P5, and P6.

In the second stage, region proposal networks^37^ slid across the multiscale pyramid feature maps extracted from the backbone net to obtain region proposals that contained tumor lesions. A window slid over every level of the feature pyramid to get anchors. The anchors had areas of {4*4, 8*8, 16*16, 32*32, 64*64} pixels on {P6, P5, P4, P3, P2}, respectively. In addition, anchors of multiple aspect ratios {1:2, 1:1, 2:1} were applied at each level. Therefore, 15 anchors were obtained simultaneously at each sliding window location. Then the anchors were fed into a 3*3 convolutional layer, which mapped the sliding window to a 256-dimensional vector. The 3*3 convolutional layer was followed by 2 sibling 1*1 convolutional layers, one of which is used for bounding box regression and the other for object/nonobject binary classification. Thus, the outputs of the regression layer were 4*15 coordinates for 15 boxes and the outputs of the classification layer were 2*15 scores for probability estimation of whether the box represented an object. Then the anchors were selected as proposals according to the Intersection-over-Union ratios of the anchors and ground-truth boxes.

In the third stage, the feature map of any region proposal was transformed into fixed spatial dimensions (7*7 for prognosis classification, molecular prediction, and bounding-box regression while 14*14 for tumor segmentation) by the RoIAlign^25^ method. Then following 3 branches were constructed for prognosis classification, molecular prediction, and mask segmentation. For the prognosis or molecular branch, the feature map was fed into 2 concatenated fully connected layers followed by 2 sibling fully connected layers, one of which is used for bounding-box regression and the other for box classification. The outputs of the regression layer were used to refine bounding-box positions for 4 subgroups, and the classification layer separately outputted probability predictions of different classes. The category with the highest probability is the forecast subgroup. For the segmentation branch, the feature map was fed into a fully convolutional network including 4 convolutional layers followed by a transposed convolutional layer with a stride of 2 to acquire the segmented mask of each proposal from RPN.

The multitask losses were obtained by performing a weighted linear sum of the losses for each individual task in the Mask-RCNN model. Hence, we could adjust the loss weight of an auxiliary task to zero and then investigate the model performance based on other tasks. The detailed network structure was described in Supplementary Text S1.

### Model Evaluation

All MB patients were divided into 2 cohorts: Firstly, 74 patients collected from Huashan Hospital and Huadong Hospital constituted the 3-fold cross-validation cohort (CVC) to assess model performance. Secondly, the patients enrolled in the previous cohort were applied to train the model and the model was performed on the remaining 39 patients from Shandong Provincial Hospital and Anhui Provincial Hospital as the independent testing cohort (ITC). Genomic characteristics, gender, OS time, and histology had no statistical differences between different evaluation cohorts (Table 1). Moreover, we researched how the 2 auxiliary tasks of prognosis categorization and tumor segmentation affected the main task performance of molecular prediction in each cohort by setting the loss weight of each auxiliary task to zero.

### Statistical Analysis

Kruskal–Wallis test analyses were performed to assess whether genomic characteristics, age at diagnosis, gender, OS time, and histology had statistical differences between different evaluation cohorts. All statistical analyses were performed by using SPSS software version 22.0 (IBM Corp.) with a 2-sided significance level of *P* value .05. In 3-fold cross-validation, the prediction probabilities and results in every fold were concatenated to assess the model performance using the accuracy (ACC) and area under the receiver operating characteristic curve (AUC) values. For prognosis prediction, we used the additional indexes of sensitivity (SEN) and specificity (SPE) to evaluate the model by treating the good prognosis subjects as positive samples and the poor prognosis subjects as negative samples. In addition, we employed a pixel-level measurement to evaluate the tumor segmentation quality of our approach: Dice coefficient. The calculation methods of quantitative indexes are described in Supplementary Text S2.

## Results

### Patient Characteristics

**Figure 2.**
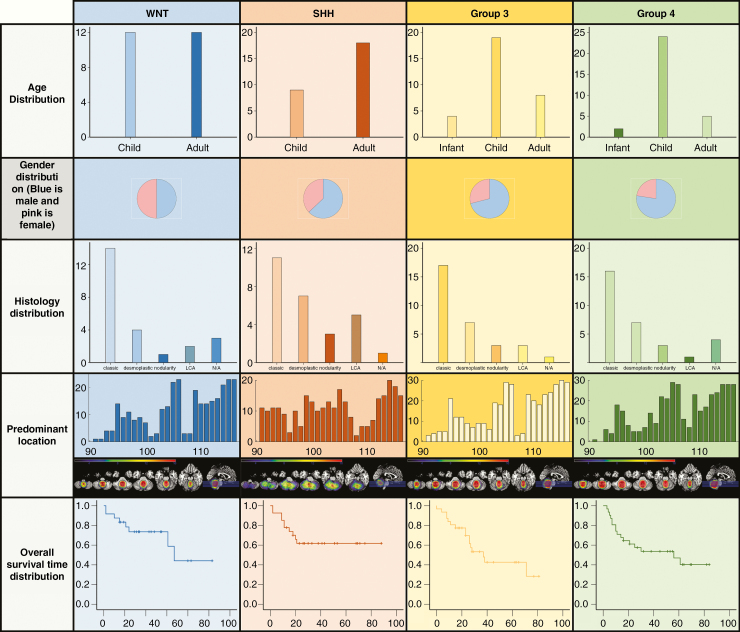
Clinical and genomic characteristics of medulloblastoma subgroups.

There were 75 male and 38 female patients enrolled in this study, consisting of 6 infant patients (mean age 4.4 years, range 3–5 years), 64 child patients (mean age 10.5 years, range 6–17 years), and 43 adult patients (mean age 30.4 years, range 18–58 years). There were 24 WNT tumors (21%), 27 SHH tumors (24%), 31 Group 3 tumors (27%), and 31 Group 4 tumors (27%). Patients’ demographic information, genetic pattern, and OS time are concluded in Figure 2. In our study cohorts, adult patients were commonly seen in the SHH subgroup. SHH tumors predominantly located in the cerebellar hemisphere were determined by a more quantitative location evaluation method (Supplementary Text S3). WNT subgroup patients were present with the most favorable clinical outcome (5-year survival rate was 80%), whereas Group 3 tumors were the worst in OS time. These results were in good accordance with previous reports.^13,38^ In our cohort, adult MB patients were presented with a trend of better survival than pediatric patients (Supplementary Figure S2). SHH tumors were predominant in the adult cohort (Supplementary Table S2).

### Evaluation of Molecular Subgrouping

**Figure 3.**
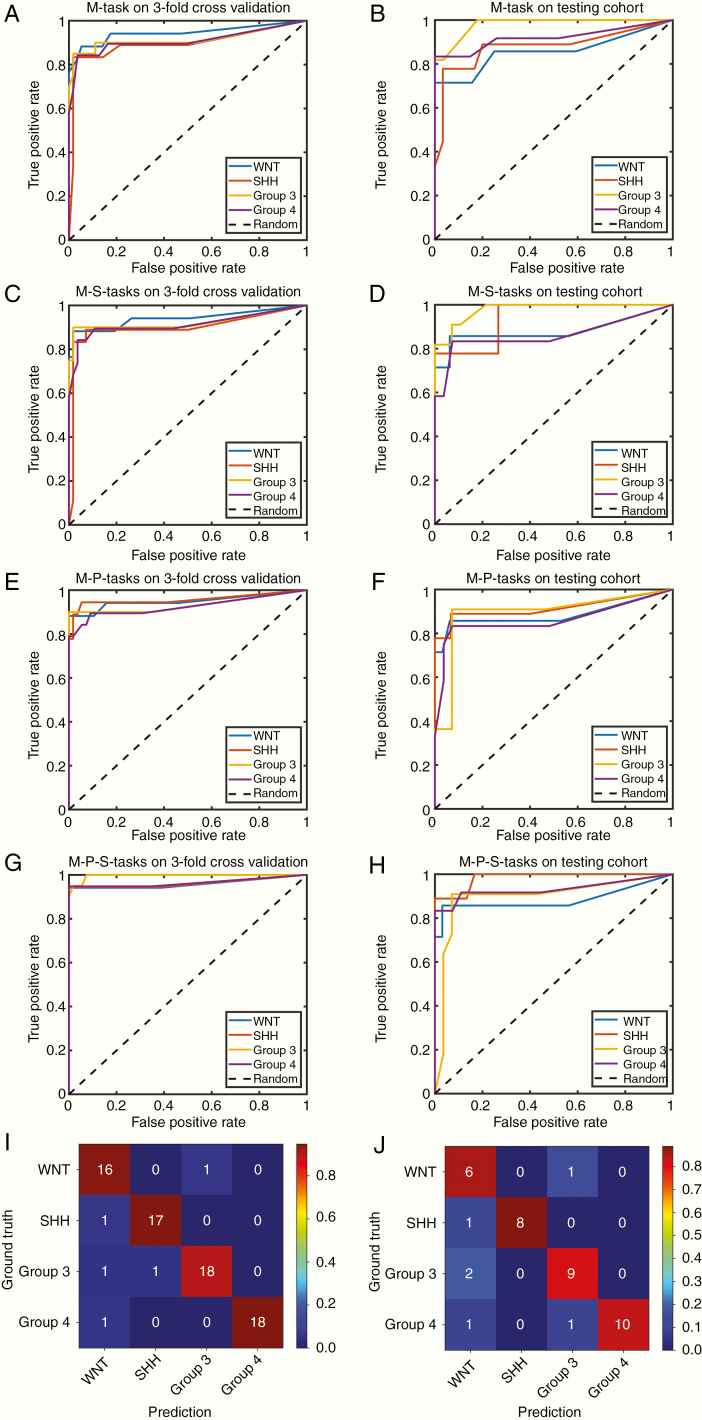
Receiver operating characteristic (ROC) curves and confusion matrices for the identification of medulloblastoma molecular subgroups with different auxiliary tasks (A and B are nonauxiliary tasks; C and D are tumor segmentation auxiliary tasks; E and F are prognosis classification auxiliary tasks; G, H, I, and J are prognosis classification and tumor segmentation auxiliary tasks). The left row figures (A, C, E, G, and I) are for the 3-fold cross-validation cohort, whereas the right row figures (B, D, F, H, and J) are for the independent testing cohort.

The evaluation results of ACC and AUC for prediction of MB subgroups are summarized in Table 2 using 3-fold CVC and ITC with different auxiliary tasks. Figure 3A–H shows the receiver operating characteristic (ROC) curves in different evaluation cohorts using different combinations of auxiliary tasks. Figure 3I–J shows the confusion matrices of the molecular prediction task assisted with prognosis classification and tumor segmentation tasks using different evaluation cohorts.

**Table 2.** Classification Results of Different Gene Subgroups With Different Auxiliary Tasks

Gene subtypes	Index	M-task^a^		M-S-tasks^b^		M-P-tasks^c^		M-P-S-tasks^d^	
		CVC	ITC	CVC	ITC	CVC	ITC	CVC	ITC
WNT	ACC	0.88	0.71	0.88	0.86	0.94	0.86	0.94	0.86
	AUC	0.94	0.86	0.94	0.88	0.95	0.88	0.96	0.88
SHH	ACC	0.83	0.78	0.89	0.78	0.89	0.89	0.94	0.89
	AUC	0.89	0.88	0.90	0.94	0.96	0.91	0.96	0.98
Group 3	ACC	0.85	0.82	0.85	0.82	0.90	0.82	0.90	0.82
	AUC	0.92	0.98	0.92	0.98	0.93	0.89	0.99	0.90
Group 4	ACC	0.79	0.83	0.79	0.83	0.79	0.75	0.95	0.83
	AUC	0.91	0.92	0.91	0.86	0.92	0.86	0.96	0.93
Average	ACC	0.84	0.79	0.85	0.82	0.88	0.82	0.93	0.85
	AUC	0.92	0.91	0.92	0.92	0.94	0.89	0.97	0.92

^a^Molecular subgrouping prediction task.

^b^Molecular subgrouping prediction and tumor segmentation tasks.

^c^Molecular subgrouping prediction and prognosis classification tasks.

^d^Molecular subgrouping prediction, prognosis classification, and tumor segmentation tasks.

**Figure 4.**
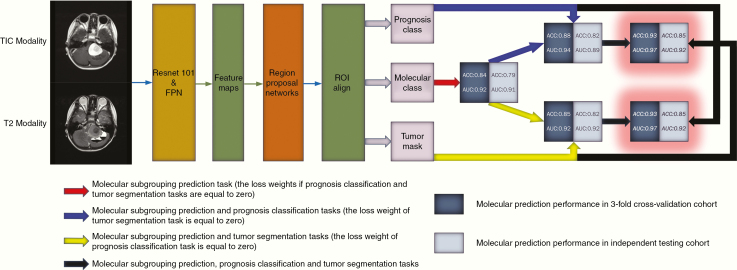
The effect of multitasking in molecular diagnosis.

The effects of multitasking are summarized in Figure 4. In the 3-fold CVC, adding an extra branch to assist in predicting molecular subtypes could improve the accuracy from 0.84 to 0.85 (added tumor segmentation branch) and further to 0.93 (added both tumor segmentation and prognosis classification branches). In the 3-fold CVC, the AUC of each molecular subgroup also improved with the assistance of prognosis classification and tumor segmentation tasks (WNT, AUC = 0.96; SHH, AUC = 0.96; Group 3, AUC = 0.99; Group 4, AUC = 0.96). Likewise, multitasking played a similar role in the ITC. Compared to the results of different cohorts, the performance dropped a little when the model was validated on the dataset from the independent institution, which demonstrated the good generalization ability and effectiveness of our proposed method in molecular prediction.

### Evaluation of Auxiliary Tasks

Patient characteristics of different prognosis categories divided according to the threshold of 27 months, which was the median OS time of all patients in our study, are given in Supplementary Table S2. Genomic characteristics, gender, and histology had no statistical differences between different OS time cohorts. Prognosis categorization was discriminated with AUC of 0.88 and 0.79, ACC of 0.80 and 0.74, SEN of 0.81 and 0.76, and SPE of 0.78 and 0.72 for 3-fold CVC and ITC, respectively (see Supplementary Figure S3 for ROC curves).

The OS time distributions of the ITC (using the true and predicted molecular subtypes) and all 113 cases are shown in Supplementary Figure S4. By comparing Supplementary Figure S4B with Supplementary Figure S4C, the predicted molecular results could provide more accurate prognosis categorization than the true molecular subtypes in the ITC.

The improved Mask-RCNN achieved an average Dice coefficient of 0.90 in both CVC and ITC in the tumor segmentation task (Supplementary Figure S5).

## Discussion

MB is an embryonal tumor with an incidence of 0.16 cases per 1 000 000 worldwide.^2,3^ Meanwhile, it is the most common malignant pediatric brain tumor with a very high rate of mortality.^1–3^ Thanks to the large-scale genomic studies of MB, it was clearly accepted that MB comprised at least 4 distinct molecular subgroups such as WNT, SHH, Group 3, and Group 4 with various stages at diagnosis, trends to metastasis, different transcriptomes patterns, copy number variations, and the clinical outcomes.^5–13^ These molecular subgroups present a critical clinical value for clinicians to designate a personal comprehensive medical paradigm for patients, especially pediatric patients. In the SJMB12 clinical trial,^39^ a workflow to treat MB patients was established and mainly depended on molecular subgroups. In Thompson et al.’s^4^ published paper, clinical data of MB patients demonstrated that the extent of resection was no more important once accounting for molecular subgroups, which would definitely change neurosurgeon’s surgical strategy. However, the methods to perform molecular subgrouping are not popularized due to technical difficulties and economic ineffectiveness. A noninvasive method for preoperative MB molecular subgrouping is ideal in the current medical situation. MRI is a clinically routine medical imaging method. Different MR findings can help clinicians make rough judgments about tumor diagnosis, surgical planning, and future prognosis. In recent years, many published articles have used MR images to determine the molecular type of MB, but most studies have been based on subjective observation, such as the location of the tumor, the enhanced morphology, and the presence or absence of cystic changes.^14–21^ For example, Perreault et al.^14^ collected 99 cases to predict molecular subgroups of pediatric MB using tumor location and enhancement pattern and achieved the accuracy of 65% in the validation cohort. The accuracy, specificity and stability of this subtype prediction method are not satisfactory. The emerging radiomics technology provides the possibility to extract the characteristics of the entire tumor by mining high-throughput features in MR images. Some researchers have applied radiomics approaches in MB for molecular diagnosis^15^; nevertheless, the diagnosis result was far from satisfaction with a limited number of cases in each molecular subgroup. Therefore, in our study, we adopted a multitask learning approach to address the few-shot problem for the accurate prediction of molecular subtyping of MB.

Multitask learning is an available solution to compensate for the lack of training data due to the low incidence of MB and molecular subtyping predisposition (lower occurrence of WNT and SHH tumors). In respect of data size, multitask learning increases the implicit data to train our models. A model that simultaneously learns multiple tasks that have different noise patterns is capable of learning a more general representation by averaging noise patterns.^26–28^ Hence, the complexity of the model and the risk of overfitting are reduced. In respect of attention focusing, if training data are limited with high-dimensional features, one task could focus its attention on those significant features as other tasks could provide additional prior knowledge to constrain model construction.^26–28^ This helps the model with improved generalization ability. In respect of feature learning, features that are difficult for one task to learn could be easy for other tasks to learn.^26–28^ The features’ eavesdropping learning mechanism makes the tasks perform better. Comparison of the prediction results presented in Table 2 shows that, although we could generally achieve acceptable performance by mainly focusing on the single task of molecular diagnosis, adding the related tasks of prognosis categorization and tumor segmentation could improve the performance of molecular prediction task significantly through the multitask learning technique. In addition, in previous studies about prognostication in MB, researchers tend to concentrate on certain age group patients such as children or adults.^40,41^ Due to the superior performance of multitasking learning, we were able to predict the prognosis for pediatric and adult patients simultaneously.

It is widely acknowledged that the clinical outcome of MB depends on not only molecular subgrouping but also on other molecular biomarkers and metastatic status.^42–44^ Hence, predicting the patient’s OS status based on a 4-type classification scheme is not adequate and could result in heterogeneous prognosis. In our study, we utilized imaging data from T1C and T2 MRI sequences to categorize the prognosis, and this method of prognosis prediction was more accurate and representative. In our study, prognostication was estimated with AUC of 0.88 and 0.79 in 3-fold CVC and ITC, respectively. In addition, the prognosis classification task provided particular prognostic information to the molecular prediction task during the establishment of the multitask framework and the outputs of molecular prediction task could reflect clearer prognostic stratification in turn compared with the true molecular categorization. As shown in Supplementary Figure S4, the predicted molecular results could provide a more accurate prognosis categorization than the true molecular subtypes.

Moreover, the variation of bioinformatics analytics makes a deep insight into intratumoral heterogeneity within 4 molecular subgroups. Based on genome-wide DNA methylation and gene expression data, Cavalli et al.^45^ worked out 12 subgroups of MB with more biological diversity, disparate prognosis, and discrepancy in response to chemo-radiotherapy. Methods based on MR images have advantages to attenuate discrepancy of intratumoral heterogeneity regarding its comprehensive insight of the tumor as a whole. For example, it is still an unresolved question for WHO 2016 consensus to completely differentiate Group 3 tumor from Group 4 tumor because of quite similar genetic background. In the study of Sharma et al.,^46^ they analyzed a series of 1501 MBs to verify that there were 8 robust Group 3/Group 4 subtypes with distinct molecular and clinic-pathological features based on large-scale methylation and expression profiling which was not easy to duplicate and apply. In this study, we could effectively distinguish Group 3 and Group 4 by the proposed method and achieve a satisfactory level of accuracy (0.90 and 0.95 corresponding to Group 3 and Group 4).

## Limitations

Preoperative MRI scans were collected from various centers using different scanner vendors and imaging parameters, with potential heterogeneity in imaging data. A multicenter, large sample data are still needed to balance the imaging data heterogeneity and demonstrate the performance of our method. There are very few infant patients in our study as the 4 hospitals involved are not children’s hospitals. Moreover, TP53 and TERTp are 2 major prognostic biomarkers beyond 4 subgroups, and we do not include them in this study because the case number will be reduced if more risk stratification is categorized. Finally, we do not have evidence of spinal metastasis of all patients. That is why we cannot use this method to predict the tendency of tumor dissemination and metastasis.

## Conclusions

With the help of multitask learning, this study represents that the MRI-based method succeeded in predicting molecular subgroups of MBs with high accuracy. Molecular subgroups were discriminated with AUC of 0.88, 0.98, 0.90, and 0.93 corresponding to WNT, SHH, Group 3, and Group 4 tumors. The final AUC of molecular diagnosis achieved 0.97 and 0.92 and prognosis subtypes were estimated with AUC of 0.88 and 0.79 in 3-fold CVC and ITC, respectively. To date, this is the first attempt at introducing multitask learning techniques into the few-shot scenario of molecular subgrouping prediction in MBs based on preoperative MRI. The high accuracy indicates that our proposed radiomic model is a promising tool that has the potential to be a useful clinical assistant.

## Supplementary Material

vdaa079_suppl_Supplementary_Material

## Notes

**Authorship statement**. X.C. and Z.F. designed the methods, carried out experiments, and wrote the paper; K.K.-W.L. and G.W. offered help in designing the methods and carrying out experiments; Z.Y., X.G., and Y.L. offered help in carrying out experiments and writing the paper; H.C. and Q.T. offered help in writing the paper and evaluating the experiments; Z.F., X.G., Y.L., and H.-K.N. acquired MR images and follow-up survey from patients; Z.S. and J.Y. proposed the project, directed the method and experiment design, and revised the paper; L.C., Y.W., Y.M., and L.Z. directed and supervised the data acquisition and follow-up survey from patients.
