# Supplementary material for: Molecular subgrouping of medulloblastoma based on few-shot learning of multitasking using conventional MR images: a retrospective multicenter study
Source: Neurooncol Adv. 2020 Jun 22;2(1):vdaa079. doi: 10.1093/noajnl/vdaa079 (PMC7393307; doi:10.1093/noajnl/vdaa079)
Supplement: vdaa079_suppl_Supplementary_Material [file vdaa079_suppl_supplementary_material.docx]

**Supplementary Tables**

**Table S1**

Table S1. MR imaging protocols

| Parameters | Institutional Cohorts | | | |
| --- | --- | --- | --- | --- |
|  | Huashan (n=52) | Huadong (n=22) | Shandong (n=16) | Anhui (n=23) |
| Manufacturer | Siemens, GE | GE | GE | Philips |
| T1C |  |  |  |  |
| TR(ms) | 300-1560 | 100-1430 | 180-1360 | 150-1000 |
| TE(ms) | 12-50 | 4-28 | 8-22 | 5-21 |
| slice thickness(mm) | 1-6 | 1-6 | 1-6 | 1-6 |
| pixel spacing(mm) | 0.4883/0.4688/0.4492 | 0.4883/0.4688/0.4492 | 0.4883/0.4688/0.4492 | 0.4883/0.4688/0.3422 |
| T2 |  |  |  |  |
| TR(ms) | 3800-8600 | 2100-6300 | 2300-5600 | 2000-7000 |
| TE(ms) | 52-146 | 40-120 | 30-100 | 35-65 |
| slice thickness(mm) | 2-6 | 2-6 | 2-6 | 2-6 |
| pixel spacing(mm) | 0.4883/0.4688/0.4492 | 0.4883/0.4688/0.4492 | 0.4883/0.4688/0.4492 | 0.4883/0.4688/0.3422 |

**Table S2**

Table S2. Patient characteristics of two datasets classified by overall survival time

|  | Overall survival time (<27m) |  | Overall survival time (>27m) | P value ^a^ |
| --- | --- | --- | --- | --- |
| **Dataset Characteristics** |  |  |  |  |
| Dataset — No. (%) | (N=55) |  | (N=58) | 0.994 |
| Cross-validation cohort | 36(65.5) |  | 38(65.5) |  |
| Independent testing cohort | 19(34.5) |  | 20(34.4) |  |
| **Clinical Characteristics** |  |  |  |  |
| Gender — No. (%) | (N=55) |  | (N=58) | 0.164 |
| Male | 15(27.3) |  | 23(39.7) |  |
| Female | 40(72.7) |  | 35(60.3) |  |
| **Gene Classification** — No. (%) | (N=55) |  | (N=58) | 0.611 |
| WNT | 9(16.4) |  | 15(25.9) |  |
| SHH | 15(27.3) |  | 12(20.7) |  |
| Group3 | 16(29.0) |  | 15(25.9) |  |
| Group4 | 15(27.3) |  | 16(27.5) |  |
| **Pathology** — No. (%) | (N=55) |  | (N=58) | 0.438 |
| Classic | 32(58.2) |  | 26(44.8) |  |
| Desmoplastic | 11(20.0) |  | 14(24.1) |  |
| Extensive nodularity | 5(9.1) |  | 5(8.6) |  |
| Anaplastic medulloblastoma | 5(9.1) |  | 6(10.3) |  |
| N/A^c^ | 2(3.6) |  | 7(12.2) |  |

^a^ P values are for the comparison between different image signature groups. Values were calculated with the use of the chi-square test.

^b^ Statistical significance.

^c^ Pathologic categories of nine patients are difficult to judge due to the suboptimal quality of tumor tissues and denoted as N/A.

**Supplementary Texts**

**Text S1**

*Feature Extraction*

In the first stage, a feature pyramid network (FPN) with the refined basic feature extraction layers of Residual Neural Network (ResNet101) was first utilized as the backbone of the Mask-RCNN. The Resnet101 extracted features from individual brain MR images and was comprised of five stages C1, C2, C3, C4, and C5 that included multiple layers such as convolutional, pooling, and fully connected layers to learn abstract representations of the original images. The extracted features went from low level to high level and became increasingly abstract with the deepening of the layers. It has been proved that more powerful representation of MR images could be obtained by using pyramid representations to construct feature pyramids based on the inherent multi-scale, pyramidal hierarchy of deep convolutional networks. Hence, multi-scale features were extracted combined the FPN with the ResNet101 in the study. The number of ResNet101 was equal to the number of input modality.

FPN constructed three parts: a bottom-up pathway, a top-down pathway, and lateral connections. The feed-forward propagation of the ResNet101 was defined as the bottom-up pathway. As previous mentioned, the feature maps represented the original images better as the deepening of the layers. Therefore, the outputs of different stages were employed to form the feature maps of different pyramid levels. In the top-down pathway, the spatially coarser, but semantically stronger feature maps from the higher pyramid levels were upsampled by a factor of two to acquire higher resolution features. In lateral connections, the feature maps from the bottom-up pathway went through a 1*1 convolutional layer to reduce the channel dimensions of all pyramid levels and to make them have a uniform value of 256. Then the upsampled features from the top-down pathway were merged with the feature maps of same spatial size from the bottom-up pathway by element-wise addition. In the double modalities model, the upsampled features of different modalities from the top-down pathway were concatenated before merging with features from the bottom-up pathway.

Finally, each merged map was appended a 3*3 convolutional layer to reduce the aliasing effect of upsampling. The final feature maps were formed by the outputs of all the 3*3 convolutional layers and called as {P2, P3, P4, P5}x which were the same spatial sizes corresponding to the outputs of stages {C2, C3, C4, C5} of ResNet101. The feature map of P6 was obtained by subsampling P5 with stride of two. The pyramid feature maps were composed of P2, P3, P4, P5, and P6.

*Region Proposal Network*

In the second stage, region proposal networks (RPN) were slid across the multi-scale pyramid feature maps from the backbone net to obtain region proposals. A window slid over every level of the feature pyramid to get anchors. The anchors had areas of {4*4, 8*8, 16*16, 32*32, 64*64} pixels on {P6, P5, P4, P3, P2} respectively. Besides, anchors of multiple aspect ratios {1:2, 1:1, 2:1} were applied at each level. Therefore, fifteen anchors were obtained simultaneously at each sliding window location. Then the anchors were fed into a 3*3 convolutional layer, which mapped the sliding window to a 256-dimensional vector. The 3*3 convolutional layer was followed by two sibling 1*1 convolutional layers, one of which is used for bounding box regression and the other for object/non-object binary classification. Thus, the outputs of regression layer were 4*15 coordinates for 15 boxes and the outputs of classificaion layer were 2*15 scores for probability estimation of whether the box represented an object. Then the anchors were selected as proposals according to the Intersection-over-Union (IoU) ratios of the anchors and ground-truth (GT) boxes. The archor which had the highest IoU for a given ground-truth box or an IoU over 0.7 with any ground-truth box was assigned a positive label while the anchor which had IoU lower than 0.3 for all ground-truth boxes was set as a negative label. Then the region proposal network was trained by the positive and negative samples and the outputs were defined as proposals.

*Classification and Segmentation*

In the third stage, the feature map of any region proposal was transformed into fixed spatial dimensions (7*7 for prognosis classification, molecular prediction and bounding-box regression while 14*14 for tumor segmentation) by the RoIAlign method. Then three followed branches determined by the number of tasks were constructed for prognosis classification/bounding-box regression, molecular prediction/bounding-box regression and mask segmentation.

For the prognosis or molecular branch, the feature map was fed into two concatenated fully connected layers followed by two sibling fully connected layers, one of which is used for bounding box regression and the other for box classification. The outputs of regression layer were used to refine bounding-box positions for four subgroups, and the classification layer separately outputted probability predictions of different classes. The category with the highest probability is the forecast subgroup. For the segmentation branch, the feature map was fed into a Fully Convolutional Network (FCN) including four convolutional layers followed by a transposed convolutional layer with stride of two to acquire the segmented mask of each proposal from RPN.

**Text S2**

The dice coefficient was calculated as following:

$$Dice= \frac{2*TP}{FP+FN+2*TP}$$

TP was the number of correctly detected pixels, FP was the number of detected pixels that were not in the ground-truth, and FN was the number of pixels in the ground-truth that were not detected.

Accuracy (ACC), sensitivity (SENS) and specificity (SPEC) for prognosis classification were calculated as following:

$$ACC=\frac{TP+TN}{TP+TN+FP+FN}$$

$$SENS=\frac{TP}{TP+FN}$$

$$SPEC=\frac{TN}{TN+FP}$$

TP, TN, FP and FN were defined as true positive, true negative, false positive and false negative respectively. The good prognosis subjects were treated as positive samples and the poor prognosis subjects were treated as negative samples.

**Text S3**

To reduce the anatomical variability among individual brain, Statistical Parametric Mapping package (SPM) was utilized to normalize the MR images into the Montreal Neurological Institute(MNI) space. The MNI brain atlas was then divided into 116 anatomical volumes of interest (AVOIs) by Anatomical Automatic Labeling (AAL). Twenty-six regions belonging to cerebellar structures were selected for analysis. After brain MR images of 113 patients were spatially registered and normalized into MNI space, the proportion of tumor tissue in each cerebellar region to the corresponding region was calculated. The proportion variable greater than 0.25 was set to one otherwise set to zero. Then a 26-dimensional location vector containing only zero and one was obtained for each individual case. The location vectors of patients in each molecular subgroup were superimposed respectively to obtain the corresponding location description statistics which could be depicted with a histogram. Besides, tumor masks of each case were applied with estimated warps generated by MR images normalization previously to acquire normalized masks. Then the masks of patients in each molecular subgroup were superimposed respectively and overlapped the Colin Holmes template to generate tumor probability distribution map. The flowchart was shown in Supplementary Figure S1 taking the WNT subtype as an example. The abscissa in the column chart represents region numbers in AAL atlas.

**Supplementary Figures**

**Figure S1**


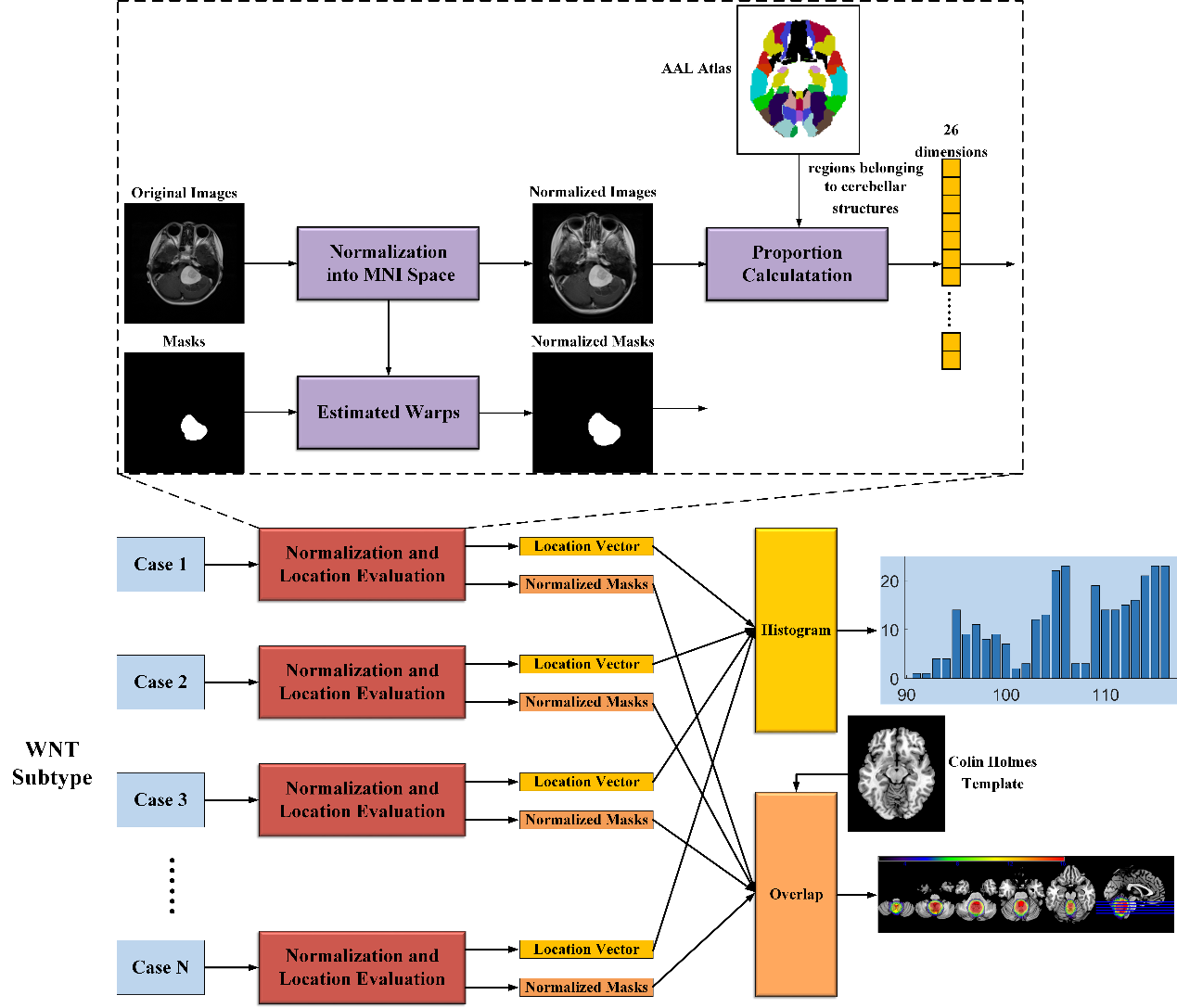


Figure S1. The flowchart of a quantitative location evaluation method

**Figure S2**


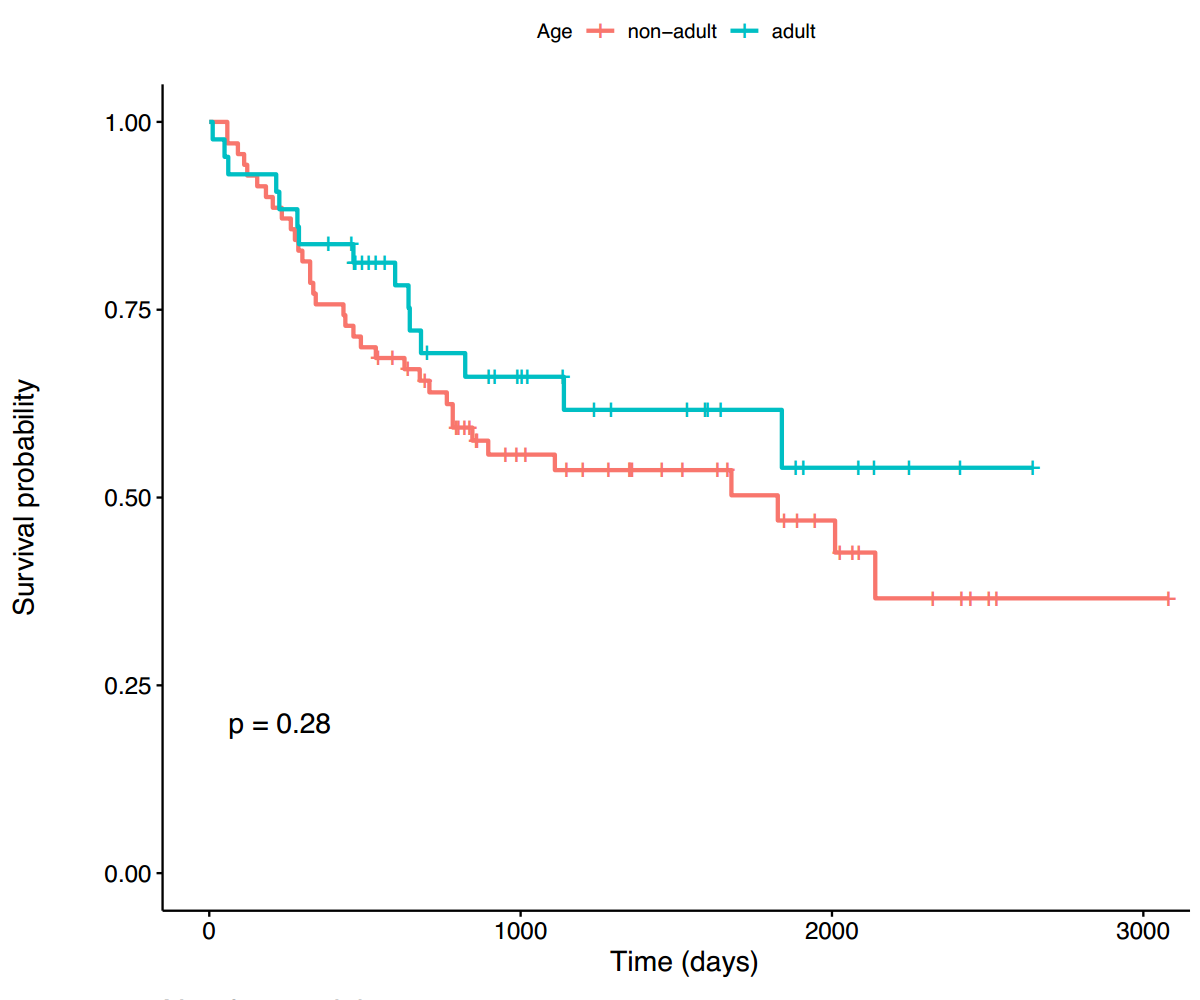


Figure S2. Prognosis of medullablastoma between different age group. Survival curves of the non-adult and adult groups were plotted using the Kaplan–Meier method. P value was calculated by log-rank test.

**Figure S3**


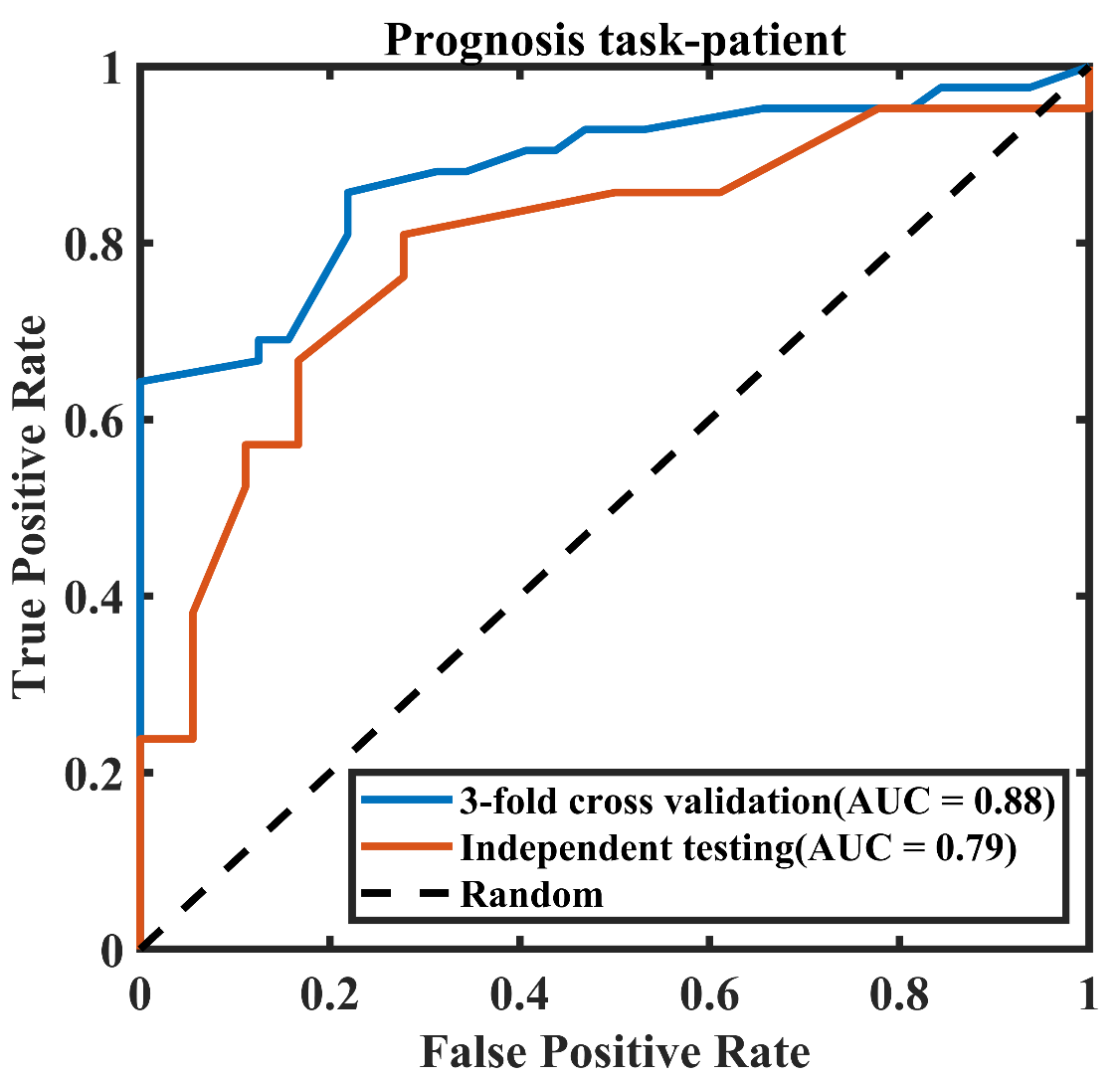


Figure S3. ROC curves for two evaluation cohorts

**Figure S4**


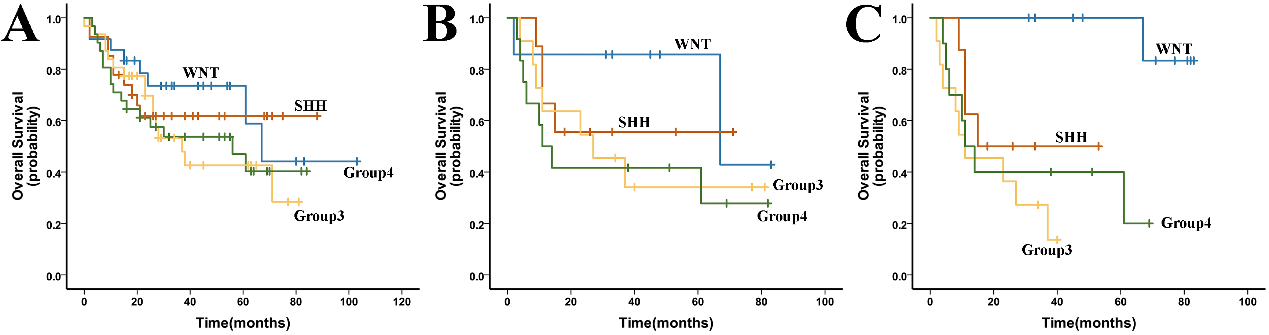


Figure S4. OS time distributions of different cohorts. (A) OS time distribution of all 113 patients: molecular subtypes; (B) OS time distribution of independent testing cohorts: true molecular subtypes; (C) OS time distribution of independent testing cohorts: predicted molecular subtypes. Compared Supplementary Figure S6B with Supplementary Figure S6C, The predicted molecular results could category the prognosis better in the independent testing cohort, which means the model implicitly learns the relationship between molecular subgroups and prognostic information.

**Figure S5**


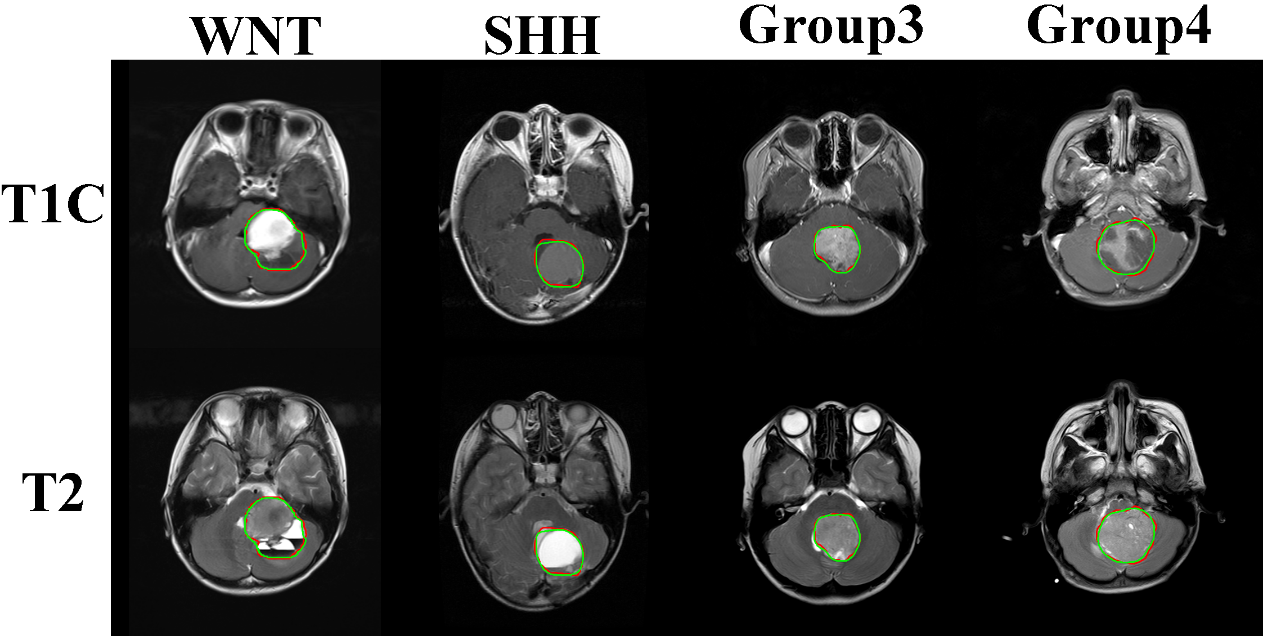


Figure S5. Tumor segmentation results of four cases from different subgroups. In each image, the red boundaries indicated the ground-truth results and the green boundaries were the results of segmentation by the Mask-RCNN within double modalities (T1C and T2) and three tasks (molecular, prognosis, and segmentation tasks).
